# First-in-class positron emission tomography tracer for the glucagon receptor

**DOI:** 10.1186/s13550-019-0482-0

**Published:** 2019-02-15

**Authors:** Irina Velikyan, Torsten Haack, Martin Bossart, Andreas Evers, Iina Laitinen, Philip Larsen, Oliver Plettenburg, Lars Johansson, Stefan Pierrou, Michael Wagner, Olof Eriksson

**Affiliations:** 1 PET Centre, Centre for Medical Imaging, Uppsala University Hospital, Uppsala, Sweden; 2 Section of Nuclear Medicine and PET, Department of Surgical Sciences, Uppsala University, Uppsala, Sweden; 3 Sanofi-Aventis Deutschland GmbH, Industriepark Höchst, 65926 Frankfurt am Main, Germany; 4 Institute of Medicinal Chemistry, Helmholtz Zentrum München, German Research Center for Environmental Health (GmbH), Neuherberg, Germany; 5 Institute of Organic Chemistry, Leibniz Universität Hannover, Hannover, Germany; 6 Antaros Medical AB, Uppsala Science Park, Dag Hammarskjölds Väg 14B, Mölndal, SE-751 83 Uppsala, Sweden; 7 Science For Life Laboratory, Department of Medicinal Chemistry, Uppsala University, Uppsala, Sweden

**Keywords:** Glucagon, GCG, GLP-1 receptor, Dual agonist, Type 2 diabetes

## Abstract

**Abstract:**

The glucagon receptor (GCGR) is emerging as an important target in anti-diabetic therapy, especially as part of the pharmacology of dual glucagon-like peptide-1/glucagon (GLP-1/GCG) receptor agonists. However, currently, there are no suitable biomarkers that reliably demonstrate GCG receptor target engagement.

**Methods:**

Two potent GCG receptor peptide agonists, S01-GCG and S02-GCG, were labeled with positron emission tomography (PET) radionuclide gallium-68. The GCG receptor binding affinity and specificity of the resulting radiopharmaceuticals [^68^Ga]Ga-DO3A-S01-GCG and [^68^Ga]Ga-DO3A-S02-GCG were evaluated in HEK-293 cells overexpressing the human GCG receptor and on frozen hepatic sections from human, non-human primate, and rat. In in vivo biodistribution, binding specificity and dosimetry were assessed in rat.

**Results:**

[^68^Ga]Ga-DO3A-S01-GCG in particular demonstrated GCG receptor-mediated binding in cells and liver tissue with affinity in the nanomolar range required for imaging. [^68^Ga]Ga-DO3A-S01-GCG binding was not blocked by co-incubation of a GLP-1 agonist. In vivo binding in rat liver was GCG receptor specific with low non-specific binding throughout the body. Moreover, the extrapolated human effective doses, predicted from rat biodistribution data, allow for repeated PET imaging potentially also in combination with GLP-1R radiopharmaceuticals.

**Conclusion:**

[^68^Ga]Ga-DO3A-S01-GCG thus constitutes a first-in-class PET tracer targeting the GCG receptor, with suitable properties for clinical development. This tool has potential to provide direct quantitative evidence of GCG receptor occupancy in humans.

**Electronic supplementary material:**

The online version of this article (10.1186/s13550-019-0482-0) contains supplementary material, which is available to authorized users.

## Introduction

Type 2 diabetes (T2D) affects hundreds of millions of individuals worldwide and causes great strain on healthcare systems globally [1]. Advancement of novel anti-diabetic treatments are therefore of utmost importance. The glucagon receptor (GCGR) is emerging as an important target in anti-diabetic therapy, especially as part of the pharmacology of dual glucagon-like peptide-1/glucagon (GLP-1/GCG) receptor agonists [2–4]. GLP1R activation has been shown to decrease blood glucose (due to the incretin effect via binding to the receptor in the pancreas), to reduce appetite and induce weight loss while agonism on the GCGR in the liver on the other hand has been shown to increase energy expenditure in addition to inducing weight loss. Thus, peptides binding both the GLP1R and GCGR are expected to combine these effects in the same molecule.

We have previously developed a target engagement marker for the glucagon-like peptide-1 receptor (GLP-1R), the positron emission tomography (PET) ligand [^68^Ga]Ga-DO3A-VS-Cys^40^-Exendin-4, which enables quantitative measurements of GLP-1R occupancy in living subjects [5–9]. Additionally, drug interaction with the GLP-1R can be assessed indirectly and qualitatively by pharmacology such as nausea. However, there is a lack of biomarkers for in vivo drug pharmacology on the GCGR, especially in the context of GLP-1R/GCGR dual agonists. Demonstrating target engagement on the GCCR remains challenging since the dual agonists activate hormone receptors with overlapping pharmacology, as well as potential cross-binding to the GLP1R.

For GLP-1/GCG receptor dual agonists, knowledge about the proportion of occupied receptors in vivo could promote the understanding of the pharmacological effects in terms of weight loss and glycemic control. The development of in vivo target engagement markers would therefore support development of dual agonists, by allowing direct measurements of drug interactions at the GCGR.

We report herein on the development and preclinical evaluation of two first-in-class PET imaging agent candidates with potential for in vivo determination of GCGR occupancy in humans.

## Materials and methods

### Peptides

The peptides S01-GCG and S02-GCG were developed by Sanofi based on exendin-4 as peptide backbone with selective amino acid mutations to achieve selectivity for the GCG receptor. Based on prior work on selective glucagon receptor agonist as rescue medicine for the treatment of severe hypoglycemia, we developed S02-GCG attaching the chelating unit DO3A via a cysteine on the c-terminus and a diethylsulfone linker. For S01-GCG, we introduced a lactam bridge between Glu16 and Lys20 to stabilize the alpha-helical fold of the peptide backbone, thereby not only increasing binding affinity and selectivity towards the GCGR but also improving metabolic stability to enhance clearance of the intact PET tracer by the kidneys. To further optimize metabolic stability, we changed the serine in position 2 to a D-serine to block against DPP-IV cleavage and mutated the arginine in position 17 to a glutamate, thereby removing the RR cleavage motif observed in glucagon. See Additional file 1 for details on peptide synthesis.

### In vitro potency assay

The potency of DO3A-S01-GCG and DO3A-S02-GCG was assessed by a functional cAMP assay in HEK293 cells transfected with human, cynomolgus non-human primate (NHP), or rat GCGR (EvoTec, Hamburg, Germany). The same assay was performed for GLP-1R to assess cross-binding. Details of the assay procedure are described in Additional file 1 and have also been described elsewhere [10].

### Chemicals

All chemicals and buffers were sourced from VWR Life Sciences, Sweden, unless otherwise noted. Peptides for blocking (glucagon, GLP-1, etc.) were synthesized in-house (Sanofi-Aventis, Frankfurt, Germany).

### Radiochemistry

The ^68^Ge/^68^Ga generator was eluted with 0.1 M HCl (3.5 mL). The pH of the eluate was adjusted to 4.6–5.0 by sodium acetate buffer (1 M, 300 μL) containing sodium hydroxide (30 μL, 10 M). To suppress the radiolysis and formation of radioactive by-products, ethanol (200 μL) as a radical scavenger was added to the reaction mixture. Then, 10 nmol of the precursor (DO3A-S01-GCG and DO3A-S02-GCG) was added. The reaction mixture was heated at 75 °C for 10–15 min. The crude product was purified using a solid phase extraction cartridge (HLB, Oasis) to assure elimination of possible hydrophilic radioactive impurities and germanium-68 and colloids. The product was eluted with 1 mL of 50% ethanol solution. The final product was formulated dependent on the biological assay.

A sample was taken for determination of radiochemical purity, peptide concentration, and pH. The total radioactivity of the product was then measured in an ionization chamber. Radiochemical purity and determination of the concentration of the peptide were determined by high-pressure liquid chromatography (HPLC). The HPLC system (LaChrom, Hitachi, VWR) consisted of an L-2130 pump, a UV detector (L-2400), and a radiation flow detector (Bioscan) coupled in series was used for product quality control. Separation of the analytes was accomplished using an endcapped analytical column with stationary reversed phase (C-4; Vaydac-C4; 50 × 4.6 mm; particle size 3 μm). The following system was used: A = 10 mM TFA; B = acetonitrile/10 mM TFA with UV detection at 220 nm; linear gradient elution 0–8 min from 24 to 44% B, 8–10 min 44% B followed by re-equilibration 10–10.5 min from 44 to 24% B and 10.5–13 min 24% B; flow rate was 1.0 mL/min. Data acquisition and handling were performed using the EZChrom Elite Software Package. The stability of the product at room temperature in 50% EtOH was monitored for 1–3 h and assessed by UV-radio-HPLC.

### Cellular internalization assay

HEK293 cells transfected with human GCGR (0.5 million cells per dish) were incubated with 3–7 nM [^68^Ga]Ga-DO3A-S01-GCG or [^68^Ga]Ga-DO3A-S02-GCG in 1 mL complete media at room temperature (RT) (5% CO_2_) for 0, 30, 60, 90, or 120 min. The assay was also performed at 4 °C (5% CO_2_) for 60 and 120 min to suppress internalization. The exact number of cells in each well was assessed by cell counter. After incubation, the hot media containing the radioligand was removed and the cells washed with serum-free media two to three times while cells were put on ice.

To measure membrane bound and internalized radioligand, cells were treated first with 0.2 M glycine buffer containing 4 M urea (pH 2.5) (acid wash buffer) and secondly 1 M NaOH (basic wash buffer).

Briefly, first 0.5 mL of acid wash buffer was added to the cells and allowed to incubate for 5 min on ice. The supernatant was then removed and measured in a well counter (membrane bound fraction) (Uppsala Imanet AB, Uppsala, Sweden). The cells were washed one time, before 0.5 mL of basic wash buffer was added and allowed to incubate for 30 min at 37 °C. Then, the detached cells were collected and measured by well counter (internalized fraction). All samples were repeated in triplicates.

The well counter data for each dish and condition was decay corrected to the time at the start of each experiment and expressed as Bq. The internalized and the total cell-associated (internalized + cell membrane bound) fractions were expressed as percentage of the total added radioligand per million of cells (%ID/M cells). The assay was repeated three times using three different batches of each radioligand.

### Cell affinity assay

GCGR-transfected cells (approximately 0.5 million cells per dish) were incubated at seven different concentrations of [^68^Ga]Ga-DO3A-S01-GCG (0.3–300 nM) or [^68^Ga]Ga-DO3A-S02-GCG (0.1–150 nM) around the expected *K*_*d*_ in 1 mL complete media for 60 min to reach steady state. The cells were incubated with radiotracer alone or in the presence of 10 μM endogenous glucagon peptide (to block GCGR and assess non-specific binding). The assay was performed at 4 °C to suppress internalization. After incubation, the cells were washed two times with complete media. For each dish, the cells were then trypsinized and re-suspended, and the supernatant was measured for cell density and radioactivity by the well counter (Uppsala Imanet AB, Uppsala, Sweden). The well counter data was decay corrected to the start of each experiment and converted to pmol bound tracer per million cells (pmol/M cells). Specific binding was determined by subtracting non-specific binding from total binding.

All samples in each assay were repeated in triplicates, and each experiment was repeated at least three times with different batches of radioligand. *K*_*d*_ and *B*_max_ for the specific binding was calculated using non-linear curve fitting in GraphPad Prism 6.05 (GraphPad, La Jolla, CA, USA).

### In vitro autoradiography assays

Liver and pancreas tissues were collected post-mortem from cynomolgus monkeys (NHP) and Sprague Dawley rats. The use of animal tissues collected post-mortem was approved by the Animal Research Ethical Committee of the Uppsala Region and was performed according to the Uppsala university guidelines on animal experimentation (UFV 2007/724).

Biopsies from donor human liver and pancreas were obtained from Uppsala Biobank (sample collection 827), and their use was approved by local ethical review board (EPN 2015/401).

Liver and pancreas frozen tissue sections (10 μm) from healthy human, NHP, and rat, as well as sectioned frozen pellets of HEK293 cells transfected with human GCGR (10 μm), were incubated with 5–10 nM [^68^Ga]Ga-DO3A-S01-GCG or [^68^Ga]Ga-DO3A-S02-GCG in 150 mL phosphate-buffered saline (PBS) (pH 7.4, 1% BSA) for 60 min at RT. The sections were incubated with radiotracer alone or together with 10 μM unlabelled precursor (S01-GCG or S02-GCG, respectively, to assess non-specific binding), 10 μM glucagon (to assess GCGR specific binding of each radiotracer), or 1 μM GLP-1 (to assess cross-binding of the radiotracers to the GLP-1R). Sections were added in duplicates in each individual assay, and each experiment was repeated at least three times with different batches of radioligand, except for rat sections that were only performed in one assay.

After incubation, the sections were washed three times in 150 mL PBS. Sections were carefully dried at 37 °C and then exposed against a digital phosphorimager plate overnight together with a 10-μl droplet of radioactive reference (cross-calibrated against a gamma counter) on an absorbent paper attached to an object glass. The phosphor imager plates were scanned using a Cyclone Plus Phosphor imager (Perkin Elmer) at 600 dpi, and the resulting autoradiograms analyzed by ImageJ software (NIH, Bethesda). Pixel values in counts/mm^2^ were converted to Bq/mm^2^ by the included reference. Bq/mm^2^ was further converted to fmol/mm^2^ by the known specific radioactivity (fmol/Bq) of each batch of radiopharmaceutical.

Additionally, affinity of [^68^Ga]Ga-DO3A-S01-GCG was assessed by in vitro autoradiography of sectioned frozen pellets of GCGR-transfected cells. The same assay conditions as above were used, unless otherwise stated. Cell pellet sections were incubated with eight different concentrations of [^68^Ga]Ga-DO3A-S01-GCG (2–150 nM). Each concentration was measured as stand-alone samples, but the entire experiment was performed twice. Non-specific binding was assessed by co-incubation with 10 μM glucagon peptide (GCG). *K*_*d*_ and *B*_max_ for the specific binding was calculated using non-linear curve fitting in GraphPad Prism 6.05.

### Rat in vivo distribution

The in vivo distribution over time was performed to identify the optimal time point for an in vivo blocking study and to calculate the residence time for dosimetry (see detail below). Thus, measurement at more time points was prioritized over repetitions at individual time points.

National and institutional guidelines for the care and use of animals were followed. All procedures performed in studies involving animals were in accordance with the ethical standards of the institution or practice at which the studies were conducted.

The in vivo organ distribution dynamics of [^68^Ga]Ga-DO3A-S01-GCG and [^68^Ga]Ga-DO3A-S02-GCG were assessed in Sprague Dawley rats (Taconic, Denmark) (327 ± 18 g, male, *n* = 16 per radioligand). The animals were kept at a constant temperature (25 °C) and humidity (50%) in a 12-h light-dark cycle. Food and water were provided ad libitum.

Each radiotracer was administered into the tail vein of conscious animals as a bolus in 0.5–0.6 mL of PBS (pH 7.4) as vehicle. Each animal received 3.9 ± 1.3 MBq/kg (3.6 ± 1.3 μg/kg) [^68^Ga]Ga-DO3A-S01-GCG or 1.7 ± 0.5 MBq/kg (1.3 ± 0.4 μg/kg) [^68^Ga]Ga-DO3A-S02-GCG radioactivity corresponding to 2–3 μg/kg peptide mass. Given the constraints of the specific radioactivity for each radioligand, the dosing was designed to minimize the given peptide mass (in order to stay below mass effect levels) while still yielding enough radioactive signal for well counter measurements.

At pre-determined post injection time points (5, 10, 20, 40, 60, 90, 120, and 180 min), two animals per each point were euthanized by CO_2_. Tissues were immediately extracted and weighed, and the radioactive content was measured in the well counter. The harvested organs were the blood, heart, lung, liver, pancreas, spleen, adrenal, kidney, small intestine (without/with its content), large intestine (without its content), feces, urinary bladder (rinsed), testis/ovary, muscle, bone, bone marrow, thyroid, and brain. The well counter readings were decay corrected to the time of injection and the mass of the extracted tissues and expressed as Bq/cc. The measurements were normalized to unitless standardized uptake values (SUV) according to Eq. 1, to allow for direct comparison between the two radiotracers.

1 $$ \mathrm{SUV}\;\left(\frac{1}{1}\right)=\frac{\raisebox{1ex}{${\mathrm{Radioactivity}}_{\mathrm{tissue}}\left({\mathrm{B}}_{\mathrm{q}}\right)$}\!\left/ \!\raisebox{-1ex}{${\mathrm{Weight}}_{\mathrm{tissue}}\left(\mathrm{g}\right)$}\right.}{\raisebox{1ex}{${\mathrm{Radioactivity}}_{\mathrm{injected}}\left({\mathrm{B}}_{\mathrm{q}}\right)$}\!\left/ \!\raisebox{-1ex}{${\mathrm{Weight}}_{\mathrm{body}}\left(\mathrm{g}\right)$}\right.} $$

### Rat in vivo competition

A competition study with each respective radiotracer precursor peptide was performed to investigate in vivo selectivity in selected tissues (the blood, heart, lung, liver, pancreas, spleen, kidney, muscle, and bone marrow). [^68^Ga]Ga-DO3A-S01-GCG or [^68^Ga]Ga-DO3A-S02-GCG were administered to conscious Sprague Dawley rats (Taconic, Denmark) (283 ± 7 g, male, *n* = 8 per radioligand). [^68^Ga]Ga-DO3A-S02-GCG (8.6 ± 1.7 MBq/kg corresponding to 6.4 ± 1.3 μg/kg) was administered in a higher radioactive and mass dose than [^68^Ga]Ga-DO3A-S01-GCG (1.9 ± 0.7 MBq/kg corresponding to 1.4 ± 0.5 μg/kg), in order to increase the relatively low detectable radioactive signal observed in the liver in the biodistribution study.

Animals in each group (*n* = 4 for each radioligand) were co-injected with 1 mg/kg of either unlabelled DO3A-S01-GCG or -DO3A-S02-GCG peptide to determine the fraction of binding in each tissue which was receptor mediated.

The animals were euthanized 40 or 60 min after administration of [^68^Ga]Ga-DO3A-S01-GCG or [^68^Ga]Ga-DO3A-S02-GCG, respectively. The time point of euthanasia for each radiotracer was determined based on liver-to-blood ratio obtained from the biodistribution study above, as well as the radionuclide half-life and known radiotracer precursor half-life in blood plasma. Tissue resection and analysis of radioactive uptake in each tissue (expressed as SUV) were performed and analyzed as described above.

### Human predicted dosimetry

The predicted dosimetry of [^68^Ga]Ga-DO3A-S01-GCG or [^68^Ga]Ga-DO3A-S02-GCG in human males was estimated based on the results from the rat in vivo biodistribution described above. The dosimetry calculations were performed as described previously for [^68^Ga]Ga-DO3A-VS-Cys^40^-Exendin4 [11].

Briefly, animal-derived tissue uptake was normalized to that of human tissues (using tissue weights of whole body adult reference male and female phantoms). The normalized SUVs were then un-decay corrected to their respective time point to reflect the actual radiation burden in each tissue. The tissue residence times (MBq-h/MBq) were assessed by trapezoidal approximation of the un-decay-corrected human SUV biodistribution data. The tissue washout from the last time point (180 min) to infinity was estimated by a single mono-exponential fit.

The estimation of the absorbed dose was performed by the OLINDA/EXM 1.1 software (Vanderbilt University, USA) where the calculations were based on the adult reference male or female phantoms to obtain the intended absorbed dose estimate in humans (ICRP60). The organ-specific doses are reported as mGy/MBq (effective dose as mSv/MBq). The amount of MBq that can be safely administered annually (MBq/year) was calculated for each organ as well as the effective dose, by dividing the limiting dose (10 mSv/year for the effective dose, 150 mGy/year for all tissues except for the red marrow and uterus with 50 mGy/year) by the dose (mGy/MBq or mSv/MBq).

### Statistics

Data on group level are reported as mean ± SEM. Statistical analysis was performed in GraphPad Prism 6.05 (GraphPad, La Jolla, CA, USA), and differences were assessed by Student’s *t* test using a significance level of *P* < 0.05.

## Results

### Potencies for the GCGR and GLP-1R

DO3A-S01-GCG and DO3A-S02-GCG activated the human, NHP, and rat GCGR with potencies in the picomolar range (Table 1). The potency at the human GCGR was comparable to that of the native glucagon. Both DO3A-S01-GCG and DO3A-S02-GCG exhibited > 50-fold selectivity than glucagon for activating GLP-1R.

**Table 1:** Potencies of S01-GCG and S02-GCG at the GCGR and GLP-1R in transfected HEK293 cells

Peptide	Human		Cynomolgus monkey		Rat	
	GCGR (pM)	GLP1R (pM)	GCGR (pM)	GLP1R (pM)	GCGR (pM)	GLP1R (pM)
DO3A-S01-GCG	0.4	19,734	1.2	4812	11.2	16,884
DO3A-S02-GCG	0.8	4611	7.9	1980	47.6	3960
Native GCG	0.5	28	1.2	31	7.5	39

All values are EC_50_ given as pM

### Radiochemistry

**Fig. 1:**
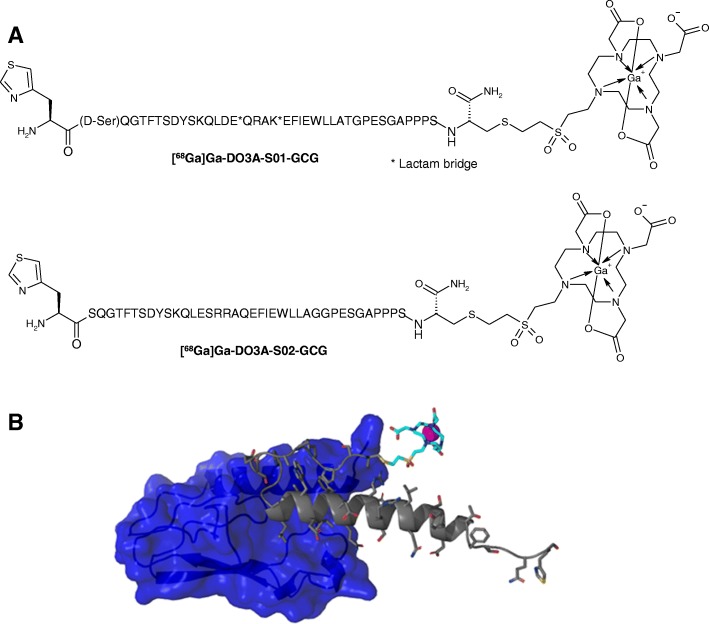
Chemical formulae of [^68^Ga]Ga-DO3A-S01-GCG and [^68^Ga]Ga-DO3A-S02-GCG depicting the overall construct similarity with difference in the peptide sequence (**a**). Three-dimensional representation of predicted interaction between [^68^Ga]Ga-DO3A-S02-GCG (gray peptide, cyan chelator, purple Ga(III), and blue GCGR) (**b**)

Reliable and highly reproducible labelling synthesis with control over the product peptide concentration and radioactivity was developed and could be accomplished within 1 h. The eluate fractionation method was modified and used for the production [12]. The parameters, time, temperature, radical scavenger, buffer concentration, pH, and product purification step, were investigated and optimized using DO3A-S02-GCG and then applied to DO3A-S01-GCG. The highest radiochemical yield was obtained at 75 °C within 10–15 min. The concentration of acetate buffer was investigated in the range of 0.01–1 M with the highest radiochemical yield and robust production obtained at 0.04 M. The optimal pH value was found to be 4.2–4.6. A number of solid phase extraction cartridges with reversed phase were tested for the purification of the crude product. The highest peptide recovery of > 96% was found for hydrophilic-lipophilic-balanced cartridges. The pure product (Fig. 1) was eluted with 1 ml of 50% EtOH and formulated in either isotonic sodium chloride or phosphate-buffered saline. If required by the biological assay, the formulated product was also sterile filtered into a sterile 10-ml capped glass bottle. Quality control was conducted using UV-radio-HPLC. Final product purification excluded contamination with ^68^Ge and colloids and provided high radiochemical purity (Table 2). The difference in radiochemical yield (RCY) and radiochemical purity (RCP) for the tracers was marginal (Table 2). The success rate (defined as RCP purity> 90% and sufficient specific radioactivity in MBq/nmol for the intended use of the batch) of the production was 100% for both imaging agents. The amount of the starting radioactivity used for the tracer production decreased with the time according to the age of the generator, however, specific radioactivity values could be kept at least around 50 MBq/nmol (Table 2) in all experiments. Addition of EtOH as a radical scavenger improved radiochemical purity.

**Table 2:** Results of ^68^Ga-labelling of DO3A-S01-GCG and DO3A-S02-GCG

Radiotracer	NDC RCY [%]	SRA @ EOS [MBq/nmol]	RA @ EOS [MBq]	RCP [%]	Amount [nmol]	Number	Success rate [%]
[^68^Ga]Ga-DO3A-S01-GCG	61.0 ± 2.0	50.2 ± 5.4	348 ± 30	98.8 ± 0.4	7.2 ± 0.5	9	100
[^68^Ga]Ga-DO3A-S02-GCG	56.9 ± 1.3	64.9 ± 2.1	471 ± 31	95.4 ± 1.4	6.96 ± 0.14	10	100

*NDC RCY* non-decay-corrected radiochemical yield, *SRA* specific radioactivity, *EOS* end of the synthesis, *RA* radioactivity, *RCP* radiochemical purity

The accurate determination of the peptide amount was accomplished by UV-HPLC (Additional file 2: Figure S1B) and assured reliability/reproducibility of the biological assays. An example of a typical UV-radio-chromatogram and UV calibration plot for [^68^Ga]Ga-DO3A-S01-GCG is given in Additional file 2: Figure S1. The stability of the tracers was investigated during 2 h with RCP of over 91% (Additional file 2: Figure S1A). The UV calibration plot covered the range of peptide concentration expected in the final imaging agent product (Additional file 2: Figure S1B).

### Cellular internalization

**Fig. 2:**
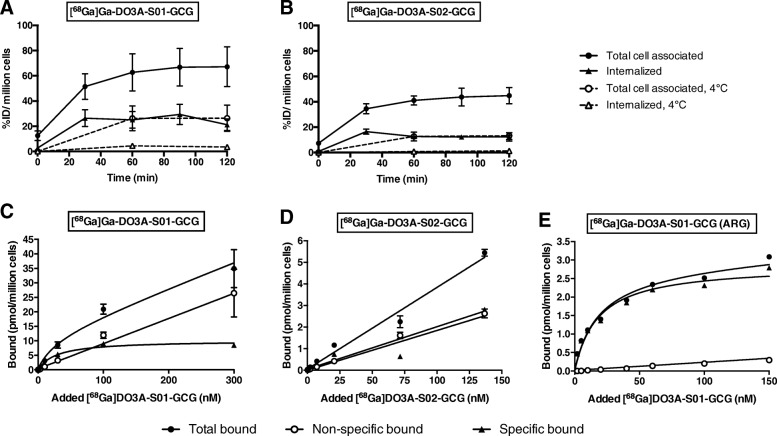
Cellular internalization and GCGR affinity of [^68^Ga]Ga-DO3A-S01-GCG and [^68^Ga]Ga-DO3A-S02-GCG. Binding and internalization in HEK293 cells overexpressing human GCGR of [^68^Ga]Ga-DO3A-S01-GCG (**a**) and [^68^Ga]Ga-DO3A-S02-GCG (**b**). Internalization was suppressed by cooling cells to 4 °C. Affinity was measured in HEK293 cells overexpressing human GCGR by saturation binding studies on viable cells (**c**, **d**) or by in vitro autoradiography (ARG) on sections of cell pellet (**e**)

[^68^Ga]Ga-DO3A-S01-GCG bound to GCGR-transfected cells reaching a steady state of approximately 65% ID/million cells after 60–120 min (Fig. 2a). The internalized fraction was 30–40% of the total binding and could be suppressed almost completely by cooling. Binding of [^68^Ga]Ga-DO3A-S02-GCG tended to be lower than that of [^68^Ga]Ga-DO3A-S01-GCG and reached a plateau of approximately 40% ID/million cells after 60 min (Fig. 2b). The internalized fraction of [^68^Ga]Ga-DO3A-S02-GCG was 30% and again suppressed by cooling.

### Affinity

[^68^Ga]Ga-DO3A-S01-GCG binding to GCGR-transfected cells could be inhibited by 10 μM GCG. Saturation binding experiments in viable cells displayed a *K*_*d*_ of 18.8 ± 10.1 nM (*n* = 3) (Fig. 2c). For [^68^Ga]Ga-DO3A-S02-GCG, it was not possible to obtain a clear saturation of binding in the range of 0.1–150 nM and the affinity was estimated to be > 100 nM (*n* = 4) (Fig. 2d). Further optimization of this value was not performed as this affinity was deemed too low to merit further exploration.

[^68^Ga]Ga-DO3A-S01-GCG saturation binding experiments of section of frozen pellets of GCGR-transfected cells yielded similar affinity as the live cell assay (*K*_*d*_ = 14.2 ± 1.0 nM (*n* = 2) (Fig. 2f).

### In vitro autoradiography

**Fig. 3:**
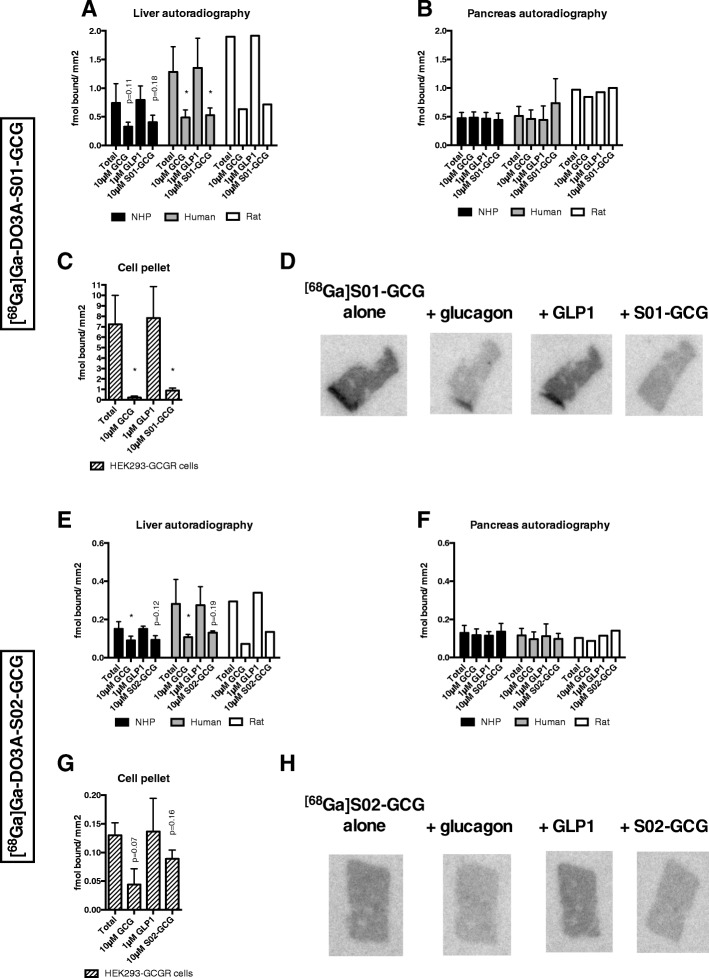
In vitro autoradiography of [^68^Ga]Ga-DO3A-S01-GCG or [^68^Ga]Ga-DO3A-S02-GCG. Binding of [^68^Ga]Ga-DO3A-S01-GCG to sections of the liver (**a**) and pancreas (**b**) from NHP (*n* = 3), human (*n* = 3), or rat (*n* = 1), or HEK293 cells overexpressing GCGR (*n* = 3) (**c**). Representative autoradiograms of [^68^Ga]Ga-DO3A-S01-GCG binding in the human liver (**d**). Binding of [^68^Ga]Ga-DO3A-S02-GCG to sections of the liver (**e**) and pancreas (**f**) from NHP (*n* = 3), human (*n* = 3), or rat (*n* = 1), or HEK293 cells overexpressing GCGR (*n* = 3) (**g**). Representative autoradiograms of [^68^Ga]Ga-DO3A-S02-GCG binding in the human liver (**h**). The sections were incubated either with radiotracer alone (Total) or together with 10 μM GCG, 1 μM GLP-1, or 10 μM of respective tracer precursor peptide S01-GCG or S02-GCG. Stars or *p* values above bars indicate difference compared to the total binding

[^68^Ga]Ga-DO3A-S01-GCG binding to liver sections in either NHP, human, or rat could be inhibited > 60% by co-incubation with GCG (Fig. 3a, d) (NHP, *n* = 3, *p* = 0.11; human, *n* = 3, *p* < 0.05; rat, *n* = 1, *p* = N/A). Co-incubation with the DO3A-S01-GCG precursor peptide in excess did not increase the degree of inhibition (NHP, *n* = 3, *p* = 0.18; human, *n* = 3, *p* < 0.05; rat, *n* = 1, *p* = N/A). Addition of GLP-1 in excess did not induce inhibition of [^68^Ga]Ga-DO3A-S01-GCG binding (NHP, *n* = 3, *p* = 0.43; human, *n* = 3, *p* = 0.35; rat, *n* = 1, *p* = N/A).

Only background binding was observed in the pancreas section regardless of species and was unaffected by the addition of either the GCG, GLP-1, or DO3A-S01-GCG precursor peptide (Fig. 3b) (NHP, *n* = 3; human, *n* = 3; rat, *n* = 1). Binding of [^68^Ga]Ga-DO3A-S01-GCG to sections of frozen pellet of GCGR-transfected cells (*n* = 3) was very strong and could be completely abolished by co-incubation with the GCG (*p* < 0.05) or DO3A-S01-GCG precursor peptide (*p* < 0.05), but not GLP-1 (Fig. 3c).

[^68^Ga]Ga-DO3A-S02-GCG binding to liver sections was lower in magnitude than [^68^Ga]Ga-DO3A-S01-GCG, but similarly inhibited by > 50% by co-incubation with the GCG (NHP, *n* = 4, *p* < 0.05; human, *n* = 3, *p* < 0.05; rat, *n* = 1, *p* = N/A) and DO3A-S02-GCG precursor peptide (NHP, *n* = 4, *p* = .012; human, *n* = 3, *p* = 0.19; rat, *n* = 1, *p* = N/A) (Fig. 3e, h). Addition of GLP-1 had no inhibitory effect on [^68^Ga]Ga-DO3A-S02-GCG binding (NHP, *n* = 4, 0.95; human, *n* = 3, *p* = 76; rat, *n* = 1, *p* = N/A). Pancreas binding was low and non-specific (Fig. 3f) (NHP, *n* = 4; human, *n* = 3; rat, *n* = 1).

Binding to GCGR-transfected cells (*n* = 3) was again lower in magnitude than for [^68^Ga]Ga-DO3A-S01-GCG and inhibited by GCG (*p* = 0.07) and DO3A-S02-GCG precursor peptide (*p* = 0.16), but not GLP-1 (Fig. 3g).

### Rat in vivo biodistribution

**Fig. 4:**
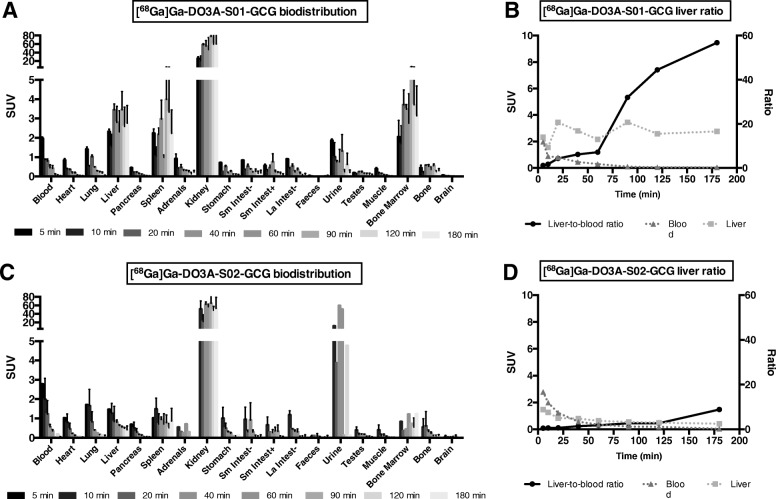
In vivo biodistribution in rat of [^68^Ga]Ga-DO3A-S01-GCG or [^68^Ga]Ga-DO3A-S02-GCG. [^68^Ga]Ga-DO3A-S01-GCG displayed rapid washout from most tissues with retention in GCGR-rich liver (**a**). The liver-to-blood ratio increased throughout 180 min (**b**). [^68^Ga]Ga-DO3A-S02-GCG also exhibited retention in the liver but to a lesser degree (**c**). Similarly, the liver-to-blood ratio was less pronounced for [^68^Ga]Ga-DO3A-S02-GCG as compared to [^68^Ga]Ga-DO3A-S01-GCG (**d**). The biodistribution data was obtained from *n* = 2 animals for each time point. The bars in panels **a** and **c** represent the average of two animals

Rapid biodistribution and subsequent washout in all tissues except the liver, spleen, kidney, and bone marrow was seen following administration of [^68^Ga]Ga-DO3A-S01-GCG (Fig. 4a and Additional file 3: Table S1). Uptake in the liver reached a plateau of approximately SUV 3 after 20 min. The liver-to-blood ratio increased continuously and was > 30 after 90 min (Fig. 4b).

Similar biodistribution observations were made for [^68^Ga]Ga-DO3A-S02-GCG, but the retention in the liver (SUV < 1), spleen, and bone marrow was lower compared to that for [^68^Ga]Ga-DO3A-S01-GCG (Fig. 4c and Additional file 4: Table S2). Accordingly, [^68^Ga]Ga-DO3A-S02-GCG liver-to-blood ratio was a modest 3 after 90 min (Fig. 4d).

### Rat in vivo competition

**Fig. 5:**
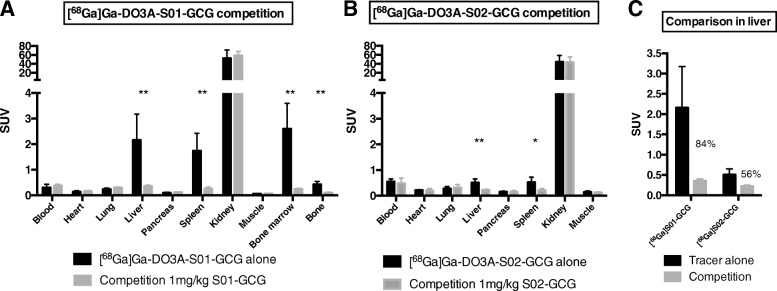
Hepatic binding of [^68^Ga]Ga-DO3A-S01-GCG and [^68^Ga]Ga-DO3A-S02-GCG can be competed away. [^68^Ga]Ga-DO3A-S01-GCG binding in the liver, spleen, and bone marrow, but not other tissues, could be competed away by co-injection with 1 mg/kg precursor peptide (**a**). Similarly, [^68^Ga]Ga-DO3A-S02-GCG binding in the liver and spleen could be competed away by 1 mg/kg precursor peptide (**b**), but to a lesser extent than for [^68^Ga]Ga-DO3A-S01-GCG (**c**). Each bar represents the average of *n* = 4 animals

[^68^Ga]Ga-DO3A-S01-GCG binding in the liver, spleen, and bone marrow was decreased by > 80% by co-injection of 1 mg/kg of the DO3A-S01-GCG precursor peptide (Fig. 5a). The high kidney uptake was not affected, nor uptake in all other examined tissues.

For [^68^Ga]Ga-DO3A-S02-GCG, binding in the liver and spleen was reduced by 56 and 60% respectively, but from a lower baseline of approximately SUV 0.5 (Fig. 5b).

Direct comparison in the liver shows that [^68^Ga]Ga-DO3A-S01-GCG has higher receptor specificity (decreased 84% by precursor peptide inhibition) as well as higher baseline binding (SUV > 2) than [^68^Ga]Ga-DO3A-S02-GCG (Fig. 5c).

### Human predicted dosimetry

**Fig. 6:**
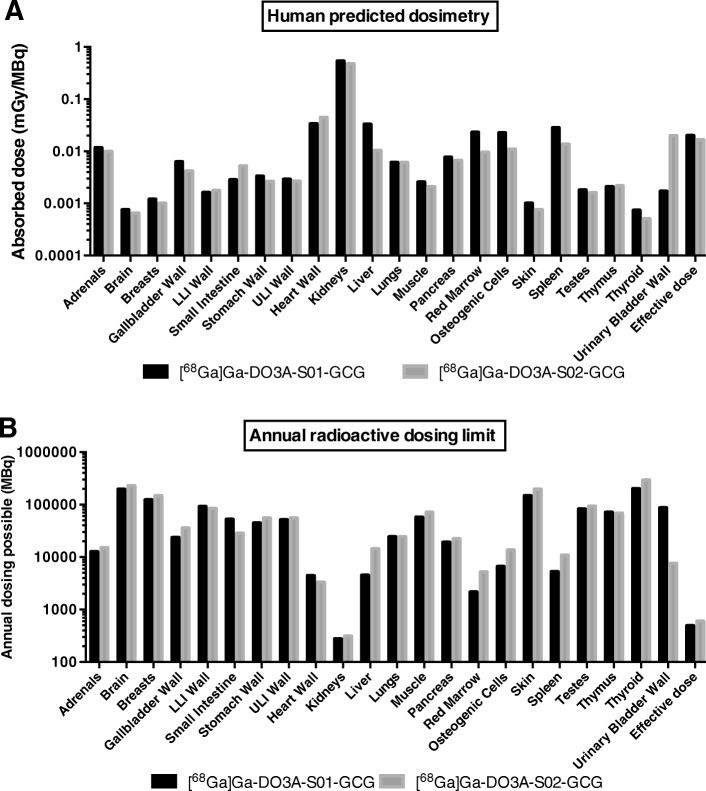
Human predicted dosimetry of [^68^Ga]Ga-DO3A-S01-GCG and [^68^Ga]Ga-DO3A-S02-GCG. The human predicted tissue-specific-absorbed dose and whole body effective dose of [^68^Ga]Ga-DO3A-S01-GCG and [^68^Ga]Ga-DO3A-S02-GCG (**a**). The maximal annual radioactive dosing possible of either radiotracer, based on the absorbed dose and the annual radiation safety limits (**b**). The renal dose was limiting for both [^68^Ga]Ga-DO3A-S01-GCG and [^68^Ga]Ga-DO3A-S02-GCG

The absorbed dose is predicted to be highest in the kidneys (0.53 and 0.48 mGy/MBq) for [^68^Ga]Ga-DO3A-S01-GCG and [^68^Ga]Ga-DO3A-S02-GCG, respectively (Fig. 6a). The kidneys were followed by the myocardium (heart wall) which also receives most of its radiation dose from the passing blood. The absorbed dose in the liver, spleen, and red marrow were somewhat higher for [^68^Ga]Ga-DO3A-S01-GCG as expected from the pronounced retention seen in the biodistribution. The whole body effective dose was also higher for [^68^Ga]Ga-DO3A-S01-GCG (20.2 μSv/MBq) compared to [^68^Ga]Ga-DO3A-S02-GCG (16.6 μSv/MBq), reflecting the generally higher binding and tissue retention.

Regarding the calculated maximal annual dosing (in MBq), the kidney dose is limiting with 278 and 312 MBq for both [^68^Ga]Ga-DO3A-S01-GCG and [^68^Ga]Ga-DO3A-S02-GCG, respectively (Fig. 6b). The whole body effective dose limits the annual exposure to 495 and 602 MBq, respectively. The bone marrow absorbed dose for [^68^Ga]Ga-DO3A-S01-GCG is predicted to allow for an excess of 2000 MBq administered annually.

## Discussion

The development of novel anti-diabetic therapies targeting glucagon receptors (GCGR) either by itself or as part of dual GLP-1/GCG agonists will profit substantially from employment of imaging techniques that enable in vivo investigation of GCGR engagement by the therapeutic agent to facilitate stratification of candidate drugs. Herein, we present radiochemistry and preclinical evaluation of two novel peptide-based analogues targeting GCGR for PET imaging that enables accurate quantification and evaluation of the target receptor occupancy.

In vitro evaluation indicated high potency of both peptide precursors for the GCGR in transfected cells, in the same range as native glucagon, but with negligible activation of the GLP-1R. The gallium-68-radiolabeled peptides exhibited strong binding to GCGR-transfected cells as well as GCGR-rich liver tissue from rat, NHP, and human. Internalization occurred as expected from an agonist, indicated to be an active process as it was suppressed at low temperatures. The binding in transfected cells as well as liver tissue was GCGR mediated, as it could be inhibited by native glucagon. Co-incubation with the precursor peptide itself did not yield any additional inhibition effect on [^68^Ga]Ga-DO3A-S01-GCG or [^68^Ga]Ga-DO3A-S02-GCG binding compared to native glucagon, indicating that both radiotracers are specific for GCGR.

Due to the structural similarity and shared transcriptional origin of the native peptides GCG and GLP-1, cross-binding to GLP-1R is a distinct possibility for any GCGR targeting agent [13]. Native glucagon for example, which was previously radiolabeled with long-lived SPECT nuclides, could potentially interact with both GCGR as well as GLP-1R, depending on exposure [14, 15]. However, careful design of the tracer compounds based on structural insights into both the GCG and GLP-1 receptor [10] led to negligible cross-binding of either radiotracer in this study as indicated by both the lack of inhibitory effect of GLP-1 on [^68^Ga]Ga-DO3A-S01-GCG or [^68^Ga]Ga-DO3A-S02-GCG binding, as well as the low baseline binding in the GLP-1R-rich pancreas.

The higher binding of [^68^Ga]Ga-DO3A-S01-GCG in cells and tissues, as compared to [^68^Ga]Ga-DO3A-S02-GCG, is likely explained by the improved affinity which was in the range of 10–20 nM regardless of the assay as well as improved pharmacokinetics (plasma *t*_½_ increased from 0.12 to 0.25 h and exposure increased from 325 to 1570 h ng/mL, data not shown). As a comparison, the affinity of [^68^Ga]Ga-DO3A-VS-Cys^40^-Exendin-4, already an established PET marker for GLP-1R receptor occupancy, has an affinity of *K*_*d*_ = 10.7 ± 3.5 nM when measured by in vitro autoradiography on human insulinoma section [16]. [^68^Ga]Ga-DO3A-S01-GCG thus has an affinity in the range suitable for quantitative in vivo PET sensing of receptor interactions.

Based on the in vitro results, strong binding and subsequent internalization of the radiotracers is expected also in vivo. After peptide digestion in the cytosol, ^68^Ga(III) remains normally in a charged state unable to passively cross the cell membrane. Thus, strong retention of ^68^Ga(III) radioactive signal is expected in tissues with GCGR expression.

Accordingly, [^68^Ga]Ga-DO3A-S01-GCG displayed an improvement compared to [^68^Ga]Ga-DO3A-S02-GCG in vivo in rat. The retention in target tissue liver was substantially higher, and the fraction that could be competed away was higher. The liver-to-blood ratio (or image contrast) was more than 30 after 90 min, indicating that the interference from the blood was minimal. Again, [^68^Ga]Ga-DO3A-S01-GCG displays similar magnitude of binding in the GCGR-rich liver as well as similar degree of receptor specificity as the established receptor occupancy PET agent [^68^Ga]Ga-DO3A-VS-Cys^40^-Exendin-4 does in the GLP1R-rich pancreas [5, 7]. In summary, the strong signal seen for [^68^Ga]Ga-DO3A-S01-GCG in the liver represents GCGR-mediated binding. This represents a large dynamic range where changes in GCGR availability—such as receptor occupancy by a candidate drug—can be quantified.

Antagonists are often preferred to agonists when developing radioligands intended for target engagement or occupancy measurement. Here, it was deemed more straightforward to develop a highly specific GCGR binding peptide agonist, due to the accumulated knowledge on the interactions of several endogenous and synthetic agonistic peptides (including native GCG, GLP-1, and Exendin4) to GCGR. In addition, the availability of an established high-throughput cell assay for potency assisted in the development of a library of agonists towards GCGR. For internalizing agonists in combination with residualizing radionuclides, as is the case here, it is suitable to consider the net rate uptake or volume of distribution as a snapshot of the receptor availability at the time of the PET examination.

The increased retention in the liver, spleen, and bone marrow for both radiotracers was receptor mediated based on the competition study. The kidney signal likely represents excretion of either radiotracer. Peptides with a mass < 60 kDa enter glomerular filtration in the kidney and may then be reabsorbed by the renal tubules and ^68^Ga(III) retained by the mechanism described above. GCGR expression has been described in the kidneys [17], but the strong excretion-mediated signal most likely masks any signal from receptor binding which explains the absence of measurable inhibition in competition studies. The binding in the spleen to a considerable extent represents receptor-mediated binding as indicated by the competition study, which is verified by previous expression studies in rat [18]. This is possibly a species-specific observation as GCGR expression has not been confirmed in the human spleen [17]. The binding to the bone marrow of [^68^Ga]Ga-DO3A-S01-GCG (Figs. 4a and 5a) and to a lesser extent [^68^Ga]Ga-DO3A-S02-GCG (Fig. 4c) is similarly likely species dependent since GCGR has not previously been reported in human. It can be speculated to be related to immune cells in rat, given the binding to both the spleen and bone marrow. The binding to the bone again represents the bone marrow, since the “bone” measurement in the assay contains the whole femur (both the cortical bone and the bone marrow).

In order to allow for measurement of GCGR occupancy of a dual GLP-1/GCG receptor agonist, the dosimetry must allow for at least two repeated PET examinations in an individual (i.e., scanning at baseline and again following drug treatment).

The predicted human dosimetry extrapolated from rat biodistribution shows that the renal dose is the limiting one for both tracers evaluated here. This is common for small peptides labeled with residualizing radionuclides such as gallium-68. When considering the possibility of repeated PET scanning, the maximal possible annual dosing (MBq) to a human individual is inversely correlated on the absorbed dose. Additionally, it depends on the regulatory safety limit set for each tissues (as described in the “Materials and methods” section above). The limiting injection dose was estimated to 278 MBq for the kidney in the case of [^68^Ga]Ga-DO3A-S01-GCG.

Assuming an administered radioactivity dose of 75 MBq per human examination, three annual PET examinations of GCGR occupancy in healthy individuals or individuals with T2D would be possible. Given that modern PET/CT scanners have improved sensitivity, combined with minimizing potential peptide mass effects on occupancy, it is likely that a radioactive dose of 50 MBq is more reasonable. This scenario would allow for up to five annual examinations of GCGR occupancy, without exceeding the radiation safety limits for the predicted kidney absorbed dose.

A similar calculation on the whole body effective dose (assuming 75 MBq [^68^Ga]Ga-DO3A-S01-GCG and an associated low-dose anatomical CT scan of 0.4 mSv) would allow for up to five PET/CT examinations. The annual scanning possible for [^68^Ga]Ga-DO3A-S02-GCG is similar as calculated for [^68^Ga]Ga-DO3A-S01-GCG. Regardless of the assumptions, the dosimetry of either radiotracer would allow for both measurement of hepatic liver occupancy during basal physiology and following drug intervention with for example a dual GLP-1/GCG receptor agonist.

In summary, [^68^Ga]Ga-DO3A-S01-GCG demonstrated favorable characteristics as a promising candidate peptide for in vivo monitoring of GCGR occupancy in humans. The possibility of quantitative GCGR PET imaging will potentially assist in pharmaceutical development as well as improved understanding of the human GCGR signaling in healthy and metabolic disease.

## Conclusion

We present evidence of first-in-class PET tracers targeting the GCG receptor. [^68^Ga]Ga-DO3A-S01-GCG has suitable properties for development as clinical PET radiotracer. This tool has potential to provide direct quantitative evidence of GCG receptor occupancy in humans.

## Additional files

Additional file 1: Supplementary materials. (DOCX 39 kb)

Additional file 2: **Figure S1.** A typical UV (blue)-Radio (red)-chromatogram of [^68^Ga]Ga-DO3A-S01-GCG at the end of the synthesis (EOS), 1 and 2 h post synthesis demonstrating stability of the imaging agent (A). UV calibration plot of [^68^Ga]Ga-DO3A-S01-GCG used for the determination of the total peptide content in the product (B). (TIF 20 kb)

Additional file 3: **Table S1.** Biodistribution of [^68^Ga]Ga-DO3A-S01-GCG over 180 min in rat. Individual values of each animal is shown (*n* = 2 per time point). (DOCX 20 kb)

Additional file 4: **Table S2.** Biodistribution of [^68^Ga]Ga-DO3A-S02-GCG over 180 min in rat. Individual values of each animal is shown (*n* = 2 per time point). * indicates failed injection in the animal. Missing value indicate that the tissue was not included in the organ list at the time of the experiment, or there were technical issues with the sampling. (DOCX 20 kb)

## Abbreviations

CT: Computed tomography
GCG: Glucagon
GCGR: Glucagon receptor
GLP-1: Glucagon-like peptide-1
GLP-1R: Glucagon-like peptide-1 receptor
HPLC: High-pressure liquid chromatography
NHP: Non-human primate
PBS: Phosphate-buffered saline
PET: Positron emission tomography
RCP: Radiochemical purity
RCY: Radiochemical yield
SUV: Standardized uptake values
T2D: Type 2 diabetes
