# Supplementary material for: First-in-class positron emission tomography tracer for the glucagon receptor
Source: EJNMMI Res. 2019 Feb 15;9:17. doi: 10.1186/s13550-019-0482-0 (PMC6377692; doi:10.1186/s13550-019-0482-0)
Supplement: Supplementary file 1 — Supplementary materials. (DOCX 39 kb) [file 13550_2019_482_MOESM1_ESM.docx]

# Supplementary material

1. *Peptide Synthesis*

*a) Synthesis of chelator VS-DO3A building block ([4,10-bis-carboxymethyl-7-(2-ethenesulfonyl-ethyl)-1,4,7,10-tetraaza-cyclododec-1-yl]-acetic acid)*

To a solution of DO3A-tBu (4,10-Bis-tert-butoxycarbonylmethyl-1,4,7,10-tetraazacyclododec1-yl)-acetic acid tert-butyl ester (2,5 g) in DMF (10 mL) was at 0 °C added a solution of divinyl sulfone (5 mL) in DMF/water 1:1 (20 mL). The mixture was allowed to reach room temperature and was stirred for 2 h. The mixture was directly purified by RP chromatography to give VS-DO3A-tBu ([4,10-Bis-tert-butoxycarbonylmethyl-7-(2-ethenesulfonyl-ethyl)-1,4,7,10-tetraazacyclododec-1-yl]-acetic acid tert-butyl ester).

A solution of VS-DO3A-tBu in TFA/water 19:1 (75 mL) was stirred at room temperature for 1 day. TFA was carefully evaporated and the remaining solution was freeze-dried to give crude VS-DO3A ([4,10-Bis-carboxymethyl-7-(2-ethene

sulfonyl-ethyl)-1,4,7,10-tetraaza-cyclododec-1-yl]-acetic acid) which was directly used without further purification.

*b) Synthesis of DO3A-S01-GCG*

For solid phase peptide synthesis Rink-Amide resin (4-(2', 4'-Dimethoxyphenyl-Fmoc-aminomethyl)-phenoxyacetamido-norleucylaminomethyl resin) was used, purchased from Novabiochem with a loading of 0.23 mmol/g, 100-200 mesh. Fmoc protected natural amino acids were purchased from Protein Technologies Inc. The following standard amino acids were used throughout the synthesis: Fmoc-L-Ala-OH, Fmoc-L-Arg(Pbf)-OH, Fmoc-L-Asp(OtBu)-OH, Fmoc-L-Gln(Trt)-OH, Fmoc-L-Glu(OtBu)-OH, Fmoc-Gly-OH, Fmoc-L-lle-OH, Fmoc-L-Leu-OH, Fmoc-L-Lys(Boc)-OH, Fmoc-L-Phe-OH, Fmoc-L-Pro-OH, Fmoc-L-Ser(tBu)-OH, Fmoc-L-Thr(tBu)-OH, Fmoc-L-Trp(Boc)-OH, Fmoc-L-Tyr(tBu)-OH, Fmoc-L-Cys(Trt)-OH. In addition, Boc-L-Tza-OH, Fmoc-DSer(tBu)-OH, Fmoc-Glu(OAll)-OH (position 16) and Fmoc-L-Lys(Alloc)-OH (position 20) were used from CSPS Pharmaceuticals and Chem-Impex International respectively.

The solid phase peptide synthesis was performed on a Prelude Peptide synthesizer (Protein Technologies Inc) using standard Fmoc chemistry and HBTU/DIPEA activation. DMF was used as the solvent. Deprotection: 20% piperidine/DMF for 2 x 2.5 min. Washes: 7 x DMF. Coupling 2:5:10 200 mM AA/500 mM HBTU/2M DIPEA in DMF 2 x for 20 min. Washes: 5 x DMF.

The crude resin was treated with phenylsilane (444µl) in 1ml DCM for 5 min. Then Pd(PPh_3_)_4_ (52mg) in 3ml DCM was added and the mixture shaken for another 40 min. The solvent was removed and the procedure repeated once. Then the resin was washed with 5ml DCM (1x5min.), 4ml sodium diethyldithiocarbamate (0.5% in DMF) (1x5min.), 4ml DMF (6x1min.) and 4ml DCM (6x1min.). The resin was treated with 165mg PyAOP, 92mg HOBT und 67µl NMM in 5ml DMF and shaken overnight at RT. The solvent was removed and the resin washed with 6x 5ml DMF and 6x 5ml DCM and dried under vacuum.

The peptide was cleaved from the resin with King's cocktail (D. S. King, C. G. Fields, G. B. Fields, Int. J. Peptide Protein Res. 36, 1990, 255-266). The crude product was purified via preparative HPLC on a Waters column (Sunfire, Prep C18) using an acetonitrile/water gradient (both buffers with 0.1%TFA). Finally, the molecular mass of the purified peptide was confirmed by LC-MS.

The purified peptide (46 mg) was then dissolved in pH 7 buffer and the solution was charged with the VS-DO3A building block (12 mg). The pH was readjusted to pH 7 using pH 10 buffer. The solution was stirred at room temperature for 16 h and was then acidified to pH 4 using acetic acid. The crude product was purified via preparative HPLC on a Waters column (Sunfire, Prep C18) using an acetonitrile/water gradient (both buffers with 0.1% formic acid). Finally, the molecular mass of the purified peptide was confirmed by LC-MS.

*c) Synthesis of DO3A-S02-GCG*

For solid phase peptide synthesis Rink-Amide resin (4-(2', 4'-Dimethoxyphenyl-Fmoc-aminomethyl)-phenoxyacetamido-norleucylaminomethyl resin) was used, purchased from Novabiochem with a loading of 0.43 mmol/g, 100-200 mesh. Fmoc protected natural amino acids were purchased from Protein Technologies Inc. The following standard amino acids were used throughout the synthesis: Fmoc-L-Ala-OH, Fmoc-L-Arg(Pbf)-OH, Fmoc-L-Asp(OtBu)-OH, Fmoc-L-Gln(Trt)-OH, Fmoc-L-Glu(OtBu)-OH, Fmoc-Gly-OH, Fmoc-L-lle-OH, Fmoc-L-Leu-OH, Fmoc-L-Lys(Boc)-OH, Fmoc-L-Phe-OH, Fmoc-L-Pro-OH, Fmoc-L-Ser(tBu)-OH, Fmoc-L-Thr(tBu)-OH, Fmoc-L-Trp(Boc)-OH, Fmoc-L-Tyr(tBu)-OH, Fmoc-L-Cys(Trt)-OH. In addition, Boc-L-Tza-OH was used from CSPS Pharmaceuticals.

The solid phase peptide synthesis was performed on a Prelude Peptide synthesizer (Protein Technologies Inc) using standard Fmoc chemistry and HBTU/DIPEA activation. DMF was used as the solvent. Deprotection: 20% piperidine/DMF for 2 x 2.5 min. Washes: 7 x DMF. Coupling 2:5:10 200 mM AA/500 mM HBTU/2M DIPEA in DMF 2 x for 20 min. Washes: 5 x DMF.

The peptide was cleaved from the resin with King's cocktail (D. S. King, C. G. Fields, G. B. Fields, Int. J. Peptide Protein Res. 36, 1990, 255-266). The crude product was purified via preparative HPLC on a Waters column (Sunfire, Prep C18) using an acetonitrile/water gradient (both buffers with 0.1%TFA). Finally, the molecular mass of the purified peptide was confirmed by LC-MS.

The purified peptide (200 mg) was then dissolved in pH 7 buffer and the solution was charged with the VS-DO3A building block (42 mg). The pH was readjusted to pH 7 using pH 10 buffer. The solution was stirred at room temperature for 16 h and was then acidified to pH 4 using acetic acid. The crude product was purified via preparative HPLC on a Waters column (Sunfire, Prep C18) using an acetonitrile/water gradient (both buffers with 0.1% TFA).

Finally, the molecular mass of the purified peptide was confirmed by LC-MS.

1. *In vitro potency assay on GLP-1 and glucagon receptor overexpressing HEK-293 cells*

Potencies of peptidic compounds at the GLP-1 and glucagon receptors were determined by functional assays measuring cAMP response of HEK-293 cell lines stably expressing human GLP-1 or glucagon receptor.

cAMP content of cells was determined using a kit from Cisbio Corp. (cat. no. 62AM4PEJ) based on HTRF (Homogenous Time Resolved Fluorescence). For preparation, cells were split into T175 culture flasks and grown overnight to near confluency in medium (DMEM / 10% FBS). Medium was then removed and cells washed with PBS lacking calcium and magnesium, followed by proteinase treatment with accutase (Sigma-Aldrich cat. no. A6964). Detached cells were washed and resuspended in assay buffer (1 x HBSS; 20 mM HEPES, 0.1% BSA, 2 mM IBMX) and cellular density determined. They were then diluted to 400000 cells/ml and 25 µl-aliquots dispensed into the wells of 96-well plates. For measurement, 25 µl of test compound in assay buffer was added to the wells, followed by incubation for 30 minutes at room temperature. After addition of HTRF reagents diluted in lysis buffer (kit components), the plates were incubated for 1 hr, followed by measurement of the fluorescence ratio at 665 / 616 nm. In vitro potency of agonists was quantified by determining the concentrations that caused 50% activation of maximal response (EC50).
