# Supplementary figures and images for: First-in-class positron emission tomography tracer for the glucagon receptor
Source: EJNMMI Res. 2019 Feb 15;9:17. doi: 10.1186/s13550-019-0482-0 (PMC6377692; doi:10.1186/s13550-019-0482-0)

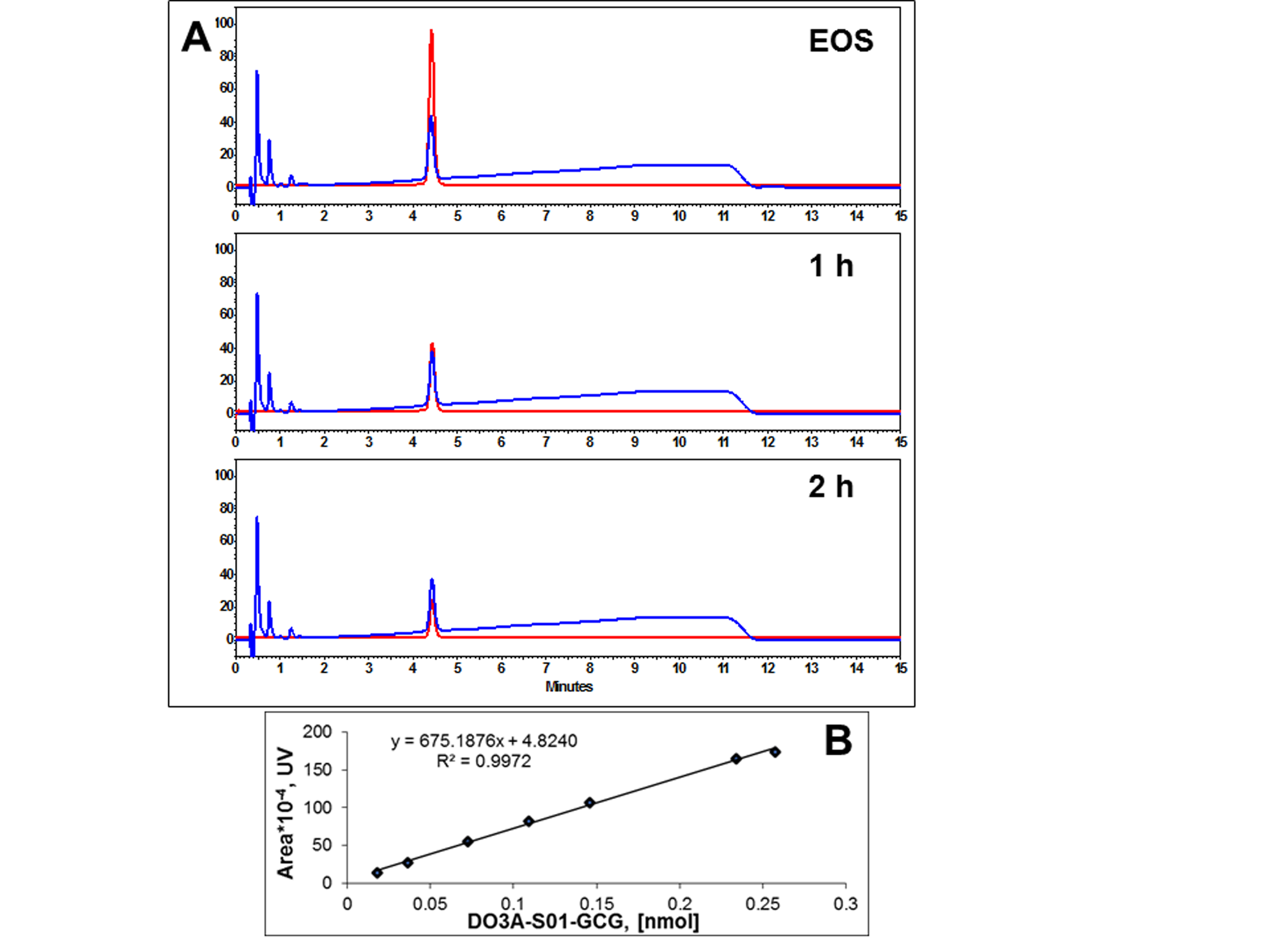

Supplement: Supplementary file 2 — Figure S1. A typical UV (blue)-Radio (red)-chromatogram of [68Ga]Ga-DO3A-S01-GCG at the end of the synthesis (EOS), 1 and 2 h post synthesis demonstrating stability of the imaging agent (A). UV calibration plot of [68Ga]Ga-DO3A-S01-GCG used for the determination of the total peptide content in the product (B). (TIF 20 kb) [file 13550_2019_482_MOESM2_ESM.tif]
