# Supplementary material for: First-in-class positron emission tomography tracer for the glucagon receptor
Source: EJNMMI Res. 2019 Feb 15;9:17. doi: 10.1186/s13550-019-0482-0 (PMC6377692; doi:10.1186/s13550-019-0482-0)
Supplement: Supplementary file 3 — Table S1. Biodistribution of [68Ga]Ga-DO3A-S01-GCG over 180 min in rat. Individual values of each animal is shown (n = 2 per time point). (DOCX 20 kb) [file 13550_2019_482_MOESM3_ESM.docx]

**Supplemental Table 1.** Biodistribution of [^68^Ga]Ga-DO3A-S01-GCG over 180 minutes in rat. Individual values of each animal is shown (n=2 per time-point).

|  | [^68^Ga]Ga-DO3A-S01-GCG | | | | | | | | | | | | | | | |
| --- | --- | --- | --- | --- | --- | --- | --- | --- | --- | --- | --- | --- | --- | --- | --- | --- |
|  | **5 min** | | **10 min** | | **20 min** | | **40 min** | | **60 min** | | **90 min** | | **120 min** | | **180 min** | |
| **Blood** | 2.04 | 1.91 | 0.9 | 0.88 | 0.69 | 0.91 | 0.31 | 0.6 | 0.56 | 0.28 | 0.07 | 0.14 | 0.03 | 0.09 | 0.04 | 0.05 |
| **Heart** | 0.92 | 0.77 | 0.42 | 0.33 | 0.32 | 0.38 | 0.11 | 0.24 | 0.22 | 0.14 | 0.06 | 0.08 | 0.02 | 0.06 | 0.03 | 0.05 |
| **Lung** | 1.53 | 1.31 | 0.56 | 0.51 | 0.92 | 1.11 | 0.48 | 0.5 | 0.31 | 0.26 | 0.26 | 0.21 | 0.12 | 0.23 | 0.05 | 0.07 |
| **Liver** | 2.45 | 2.21 | 2.18 | 0.93 | 3.14 | 3.75 | 1.98 | 3.63 | 1.24 | 3.4 | 2.51 | 4.39 | 1.58 | 3.59 | 1.85 | 3.67 |
| **Pancreas** | 0.48 | 0.43 | 0.24 | 0.21 | 0.18 | 0.25 | 0.1 | 0.17 | 0.13 | 0.1 | 0.06 | 0.06 | 0.03 | 0.06 | 0.03 | 0.04 |
| **Spleen** | 2.03 | 2.46 | 1.52 | 0.6 | 1.85 | 2.51 | 1.99 | 3.95 | 0.87 | 1.11 | 1.88 | 6.09 | 0.9 | 5.78 | 1.03 | 3.44 |
| **Adrenals** | 1.16 | 0.68 | 0.44 | 0.42 | 0.33 | 0.62 | 0.27 | 0.36 | 0.31 | 0.33 | 0.18 | 0.31 | 0.06 | 0.22 | 0.15 | 0.32 |
| **Kidney** | 22.51 | 29.84 | 32.23 | 9.44 | 56.05 | 60.71 | 42.57 | 67.98 | 19.08 | 75.17 | 54.43 | 101.42 | 33.33 | 85.4 | 27.12 | 93.19 |
| **Stomach** | 0.7 | 0.74 | 0.25 | 0.25 | 0.45 | 0.57 | 0.23 | 0.16 | 0.31 | 0.21 | 0.1 | 0.12 | 0.04 | 0.13 | 0.03 | 0.11 |
| **Sm Intest-** | 0.86 | 0.83 | 0.37 | 0.35 | 0.42 | 0.64 | 0.38 | 0.41 | 0.23 | 0.25 | 0.16 | 0.39 | 0.07 | 0.27 | 0.06 | 0.32 |
| **Sm Intest+** | 0.64 | 0.49 | 0.36 | 0.23 | 0.33 | 0.59 | 1.17 | 0.34 | 0.17 | 0.33 | 0.16 | 0.25 | 0.05 | 0.23 | 0.06 | 0.14 |
| **La Intest-** | 0.89 | 0.93 | 0.46 | 0.37 | 0.42 | 0.65 | 0.19 | 0.29 | 0.43 | 0.26 | 0.06 | 0.22 | 0.04 | 0.11 | 0.06 | 0.16 |
| **Faeces** | 0.06 | 0.01 | 0.03 | 0.01 | 0 | 0.01 | 0.01 | 0 | 0.01 | 0.02 | 0 | 0.01 | 0 | 0.04 | 0.01 | 0.07 |
| **Urine** | 1.79 | 1.95 | 0.27 | 1.76 | 0.71 | 0.82 | 0.13 | 1.4 | 2.16 | 0.47 | 0.13 | 0.36 | 0.04 | 1.21 | 0.08 | 0.34 |
| **Testes** | 0.21 | 0.18 | 0.17 | 0.07 | 0.24 | 0.26 | 0.16 | 0.28 | 0.17 | 0.15 | 0.11 | 0.17 | 0.06 | 0.17 | 0.06 | 0.12 |
| **Muscle** | 0.46 | 0.34 | 0.22 | 0.11 | 0.14 | 0.17 | 0.07 | 0.1 | 0.08 | 0.05 | 0.02 | 0.03 | 0.01 | 0.04 | 0.02 | 0.03 |
| **Bone Marrow** | 2.91 | 1.2 | 2.63 | 0.78 | 2.94 | 4.48 | 3.24 | 3.73 | 1.24 | 4.28 | 6.78 | 5.43 | 1.5 | 5.92 | 1.59 | 4.69 |
| **Bone** | 0.59 | 0.32 | 0.26 | 0.14 | 0.59 | 0.59 | 0.43 | 0.62 | 0.24 | 0.49 | 0.47 | 0.65 | 0.18 | 0.31 | 0.2 | 0.41 |
| **Brain** | 0.07 | 0.07 | 0.03 | 0.03 | 0.02 | 0.03 | 0.01 | 0.03 | 0.02 | 0.01 | 0 | 0.01 | 0 | 0.01 | 0 | 0 |
