# Supplementary material for: First-in-class positron emission tomography tracer for the glucagon receptor
Source: EJNMMI Res. 2019 Feb 15;9:17. doi: 10.1186/s13550-019-0482-0 (PMC6377692; doi:10.1186/s13550-019-0482-0)
Supplement: Supplementary file 4 — Table S2. Biodistribution of [68Ga]Ga-DO3A-S02-GCG over 180 min in rat. Individual values of each animal is shown (n = 2 per time point). * indicates failed injection in the animal. Missing value indicate that the tissue was not included in the organ list at the time of the experiment, or there were technical issues with the sampling. (DOCX 20 kb) [file 13550_2019_482_MOESM4_ESM.docx]

**Supplemental Table 2.** Biodistribution of [^68^Ga]Ga-DO3A-S02-GCG over 180 minutes in rat. Individual values of each animal is shown (n=2 per time-point). * indicates failed injection in the animal. Missing value indicate that the tissue was not included in the organ list at the time of the experiment, or there were technical issues with the sampling.

|  | [^68^Ga]Ga-DO3A-VS-S02-GCG | | | | | | | | | | | | | | | |
| --- | --- | --- | --- | --- | --- | --- | --- | --- | --- | --- | --- | --- | --- | --- | --- | --- |
|  | **5 min** | | **10 min** | | **20 min** | | **40 min** | | **60 min** | | **90 min** | | **120 min** | | **180 min** | |
| **Blood** | 2.78 | * | 2.75 | 1.22 | 1.72 | 0.74 | 0.66 | 0.48 | 0.31 | 0.4 |  | 0.02 |  | 0.18 | 0.05 | 0.04 |
| **Heart** | 1.03 | * | 1.11 | 0.48 | 0.67 | 0.24 | 0.23 | 0.22 | 0.13 | 0.12 |  | 0.04 |  | 0.07 | 0.04 | 0.02 |
| **Lung** | 1.71 | * | 2.26 | 1.06 | 1.2 | 0.44 | 0.39 | 0.41 | 0.24 | 0.24 | 0.16 | 0.16 |  | 0.13 | 0.09 | 0.13 |
| **Liver** | 1.47 | * | 1.63 | 0.9 | 1.4 | 0.28 | 0.72 | 0.89 | 0.62 | 0.66 | 0.58 | 0.51 | 0.53 | 0.47 | 0.28 | 0.56 |
| **Pancreas** | 0.69 | * | 0.72 | 0.3 | 0.42 | 0.16 | 0.18 | 0.15 | 0.15 | 0.11 | 0.05 | 0.03 | 0.04 | 0.06 | 0.05 | 0.02 |
| **Spleen** | 1.02 | * | 1.89 | 1.1 | 1.09 | 0.33 | 0.86 | 1.03 | 0.35 | 1.08 | 0.5 | 1.06 | 0.36 | 0.67 | 0.4 | 1.33 |
| **Adrenals** |  | * |  | 0.55 |  | 0.31 |  | 0.24 |  | 0.71 |  | 0.3 |  | 0.05 |  | 0.05 |
| **Kidney** |  | * | 64.75 | 38.02 | 33.32 | 11.44 | 58.36 | 66.34 | 52.52 | 60.93 | 43.66 | 86.29 | 42.02 | 54.97 | 34.72 | 71.19 |
| **Stomach** |  | * | 1.41 | 0.62 | 0.68 | 0.32 | 0.33 | 0.28 | 0.27 | 0.2 |  | 0.09 | 0.08 | 0.11 | 0.13 |  |
| **Sm Intest-** |  | * | 1.39 | 0.5 | 0.37 | 0.27 | 1.55 | 0.28 | 0.37 | 0.23 | 0.06 | 0.12 | 0.1 | 0.08 | 0.13 | 0.06 |
| **Sm Intest+** |  | * | 0.95 | 0.37 | 0.29 | 0.29 | 0.38 | 0.16 | 0.21 | 0.49 | 0.57 | 0.09 | 0.1 | 0.06 | 0.05 | 0.07 |
| **La Intest-** |  | * | 1.33 | 1.05 | 0.47 | 0.46 | 0.38 | 0.29 | 0.41 | 0.2 | 0.07 | 0.08 | 0.02 | 0.09 | 0.13 | 0.04 |
| **Faeces** |  | * | 0.11 | 0.02 | 0.11 |  | 0.18 | 0.02 | 0.07 | 0.02 | 0.01 | 0.01 | 0.04 | 0 | 0.1 | 0.01 |
| **Urine** |  | * |  | 11.7 |  | 3.87 |  | 59.55 |  | 51.22 |  | 0.13 |  | 4.78 |  | 0.12 |
| **Testes** |  | * | 0.51 | 0.28 | 0.21 | 0.14 | 0.18 | 0.19 | 0.17 | 0.19 | 0.05 | 0.08 | 0.02 | 0.09 | 0.03 | 0.05 |
| **Muscle** |  | * | 0.59 | 0.23 | 0.17 | 0.07 | 0.17 | 0.07 | 0.08 | 0.06 | 0.03 | 0.02 | 0.01 | 0.02 | 0.03 | 0.01 |
| **Bone Marrow** |  | * |  | 0.84 |  | 0.36 |  | 0.44 |  | 1.22 | 0.74 |  | 0.22 | 0.89 |  | 1.25 |
| **Bone** |  | * | 0.86 | 0.25 | 1.14 | 0.08 | 0.38 | 0.27 | 0.28 | 0.19 | 0.11 | 0.12 | 0.08 | 0.13 | 0.1 | 0.16 |
| **Brain** |  | * | 0.1 | 0 | 0.04 | 0.03 | 0.05 | 0.02 | 0.06 | 0.02 | 0.01 | 0.12 | 0 | 0.01 |  | 0.01 |
